# Evaluation of autonomic nervous system activity in intradialytic hypotension using entropy and skin sympathetic nerve activity

**DOI:** 10.3389/fnins.2023.1196750

**Published:** 2023-05-15

**Authors:** Jiayi Li, Yantao Xing, Yike Zhang, Chang Cui, Jing Wang, Jianqing Li, Chengyu Liu

**Affiliations:** ^1^State Key Laboratory of Digital Medical Engineering, School of Instrument Science and Engineering, Southeast University, Nanjing, China; ^2^Division of Cardiology, The First Affiliated Hospital of Nanjing Medical University, Nanjing, China; ^3^Division of Nephrology, The First Affiliated Hospital of Nanjing Medical University, Nanjing, China

**Keywords:** ECG, skin sympathetic nerve activity, entropy, intradialytic hypotension, heart rate variability, autonomic nervous system, hemodialysis

## Abstract

**Introduction:**

The function of the autonomic nervous system (ANS) is crucial in the development of intradialytic hypotension (IDH). This study introduced the entropy of heart rate variability (HRV) and skin sympathetic nerve activity (SKNA) to provide a complementary nonlinear and dynamic perspective for evaluating ANS function concerning IDH.

**Methods:**

93 patients undergoing hemodialysis (HD) were enrolled, and the baseline data, electrocardiogram (ECG), and SKNA were collected. The patients were separated into the IDH and nonIDH groups based on the thresholds, which were characterized as reductions in systolic blood pressure (SBP) of at least 20 mm Hg or mean arterial pressure (MAP) of at least 10 mm Hg. We developed a logistic regression model for IDH after analyzing the changes in the time domain, frequency domain, the entropy of HRV, and SKNA indices during HD.

**Results:**

After 4-h HD, the detected results for heart rate, the ratio of low frequency and high frequency (LF/HF), and average SKNA (aSKNA) all increased in both groups. Nine out of the ten HRV indices and aSKNA in the nonIDH group were higher than those in the IDH group at most moments. aSKNA was positively correlated with heart rate (*p* = 0.0001) and LF/HF (*p* = 0.0005) in the nonIDH group, while the correlation disappeared in the IDH group, which indicated a worse ANS response in IDH patients. The logistic regression model exhibited the results of initial SBP [odds ratio (OR) 1.076; *p* = 0.001], and the difference between the last and first segments (DLF) of heart rate [OR 1.101; *p* =0.012] and LF/HF [OR 0.209; *p* =0.034], as well as the extreme value of the difference between other segments and the first segments (EOF) of aSKNA [OR 2.908; *p* =0.017], which were independent indicators for IDH.

**Discussion:**

The new nonlinear and dynamic assessment perspectives provided by the entropy of HRV and SKNA help to distinguish differences in ANS patterns between IDH patients and nonIDH patients and have the potential to be used in clinical monitoring for HD patients.

## 1. Introduction

Intradialytic hypotension (IDH), as one of the main complications during hemodialysis (HD), is related to several adverse prognostic events, including inadequate dialysis dose (Ronco et al., 2000), end-organ ischemia (MacEwen et al., 2017; Seong et al., 2018), increased cardiovascular events (Stefánsson et al., 2014) and mortality (Shoji et al., 2004). It is crucial to have a throughout understanding of the physiological mechanism of IDH to effectively prevent and treat the condition.

During HD, as intravascular volume decreases, compensatory mechanisms are activated to counter the tendency to fall in blood pressure (BP) by increasing the plasma refill, cardiac output, and peripheral vascular resistance (Chou et al., 2017). IDH is the consequence of ultrafiltration exceeding plasma replacement (Davenport, 2022). The autonomic nervous system (ANS), comprising the sympathetic and parasympathetic nervous systems, is essential to this process. The sympathetic nervous activity (SNA), vascular resistance, and heart rate are found to increase in patients without IDH during HD, while decreasing during IDH episodes in IDH-prone patients, which indicates that insufficient sympathetic response contributes to IDH (Converse et al., 1992). Therefore, the accurate assessment of ANS patterns contributes to an in-depth understanding of IDH.

Heart rate variability (HRV) is a useful and noninvasive method for evaluating ANS function, which represents the changes in continuous heartbeats (Heart rate variability, 1996). Time and frequency domain analysis of HRV is widely utilized to assess IDH in patients with HD (Pelosi et al., 1999; Chang et al., 2016; Park et al., 2019). Nevertheless, the heart is a nonlinear dynamic system, and these linear statistical measures may mask the abnormal nonlinear information on heart rhythm (Denton et al., 1990). Entropy is used to evaluate the regularity between time intervals, where increased regularity tends to indicate a defect in the regulatory system (Mayer et al., 2014). Therefore, we introduced entropy methods to describe the relationship between regularity changes in heartbeats and ANS regulation.

Skin sympathetic nerve activity (SKNA) is a recent and high-frequency method for the noninvasive detection of SNA, which is proved to be well correlated with stellate ganglion activity and valid in related studies of diseases with abnormal SNA (Jiang et al., 2015; Doytchinova et al., 2017; He et al., 2020; Kusayama et al., 2020a). The cardiac sympathetic nerve alternates at the stellate ganglion, and its postganglionic fibers control cardiac activity. Thus stellate ganglion activity is indicative of sympathetic activity. SKNA provides a new perspective for evaluating SNA with the second-by-second temporal resolution. It can be applied to sinus node dysfunction scenarios, which are unavailable with HRV (Kusayama et al., 2020b). In an anesthesia injection study, SKNA was found to be superior to HRV in describing the inhibition of SNA (Xing et al., 2022). SKNA can be used as a more intuitive way to describe SNA, which complements the HRV’s description of the ANS function.

To research the influence of the ANS on IDH, especially the SNA, we recruited patients to compare the ANS patterns between those who experienced IDH and those who did not during HD. A wearable device was applied to conveniently and noninvasively acquire physiological signals from HD patients. To the best of our knowledge, our study is the first to implement the methods for the entropy of HRV and SKNA to provide the nonlinear and dynamic perspective of the ANS function on IDH. Besides, we conducted correlations between SKNA and HRV indices to explore the mapping relationship. Moreover, the multivariate model was established by binary logistic regression based on baseline data, HRV, and SKNA indicators to determine the risk factors of IDH.

## 2. Methods

### 2.1. Participants

The study enrolled 93 patients who underwent maintenance HD at the First Affiliated Hospital of Nanjing Medical University between August and November 2020. All participants in this study were over the age of 18 and had been receiving HD treatment for at least 3 months, with each session lasting 4 h, three times a week. Patients who had a previous history of arrhythmia, cerebrovascular disease, heart valve disease, acute coronary syndrome, pacemaker installation, severe anemia, or severe infection were excluded. Written informed consent was obtained from all subjects before they participated in the study. To protect patients’ privacy, all data were anonymized during the analysis procedure. The study was conducted after receiving approval from the Ethics Committee of the First Affiliated Hospital of Nanjing Medical University.

### 2.2. Baseline data

The baseline data were collected, including age, sex, body mass index, HD duration, ultrafiltration, the ratio of ultrafiltration and weight, systolic blood pressure (SBP) and diastolic blood pressure (DBP) before HD. The measurement of systolic blood pressure (SBP) and diastolic blood pressure (DBP) was conducted before HD, as the baseline, and every hour after the start of HD.

### 2.3. Blood pressure analysis

We used the four indicators of SBP, DBP, mean arterial pressure (MAP), and pulse pressure (PP) to comprehensively evaluate BP in HD. MAP was determined by adding 1/3 SBP and 2/3 DBP, while PP was determined as SBP minus DBP.

There is no widely accepted definition for the condition in previous studies on IDH. We defined IDH, referring to the K/DOQI Clinical Practice Guidelines (K/DOQI Clinical Practice Guidelines for Cardiovascular Disease in K/DOQI Workgroup, 2005), as a reduction in SBP of at least 20 mm Hg or a reduction in MAP of at least10 mm Hg. To be clear, we omitted the clinical symptoms in our study, compared to the definition in the guidelines. On one hand, it is possible that symptoms and treatments are unnecessarily linked to end-organ damage or hemodynamic instability, which could be deceptive (Assimon and Flythe, 2017). On the other hand, we intended to pay more attention to the changes in objective BP values to uncover the relevant physiological changes in patients with asymptomatic or latent IDH. The subjects with an SBP reduction of at least 20 mmHg or a MAP reduction of at least 10 mmHg were divided into the IDH group. Otherwise, they were divided into the nonIDH group.

### 2.4. Acquisition of ECG and SKNA

**Figure 1:**
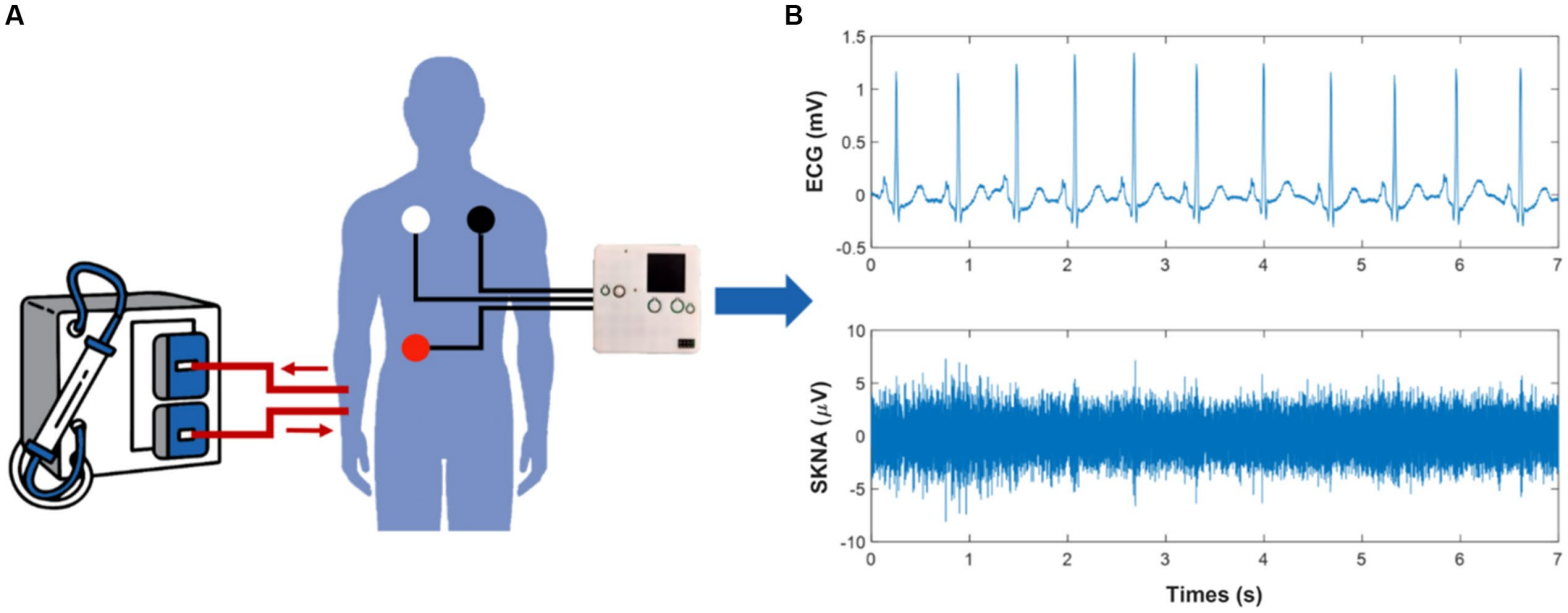
Schematic of the physiological signal acquisition process and data processing. **(A)** illustrates the scenario of signal acquisition during hemodialysis (HD). **(B)** shows the electrocardiogram (ECG) and skin sympathetic nerve activity (SKNA) signals separated from the raw signals, respectively.

Our team developed a portable, noninvasive, and high-frequency electrophysiological signal acquisition device with a sampling frequency of up to 16 kHZ, input noise as low as 0.1μV_rms_, and size of 7 mm * 8 mm * 2 mm, which can simultaneously collect ECG and SKNA signals (Xing et al., 2022). Before this, SKNA had not been acquired by proprietary acquisition equipment. In comparison with the reference system, the acquired signal quality of the device was verified to be effective and reliable (Xing et al., 2020, 2022). In this study, we used this device to acquire physiological signals from HD patients. Subjects were required to remain in the supine position and avoid unnecessary movement during HD to improve signal quality, while ECG and SKNA signals were simultaneously measured by the devices for 4 h (Figure 1A). The sampling frequency was 4 kHz. Three wet electrodes were applied to the skin of each subject to monitor single lead signals.

### 2.5. Preprocessing of ECG and SKNA

ECG and SKNA signals were obtained through a 150 Hz low-pass filter and a 500 to 1,000 Hz band-pass filter, respectively (Figure 1B). We introduced the preprocessing processes of noise reduction (Pandit et al., 2017), R-peak detection (Wang et al., 2022), and signal quality assessment (Liu et al., 2019) to ensure the reliability of RR intervals. Furthermore, RR intervals that changed more than 20% from the previous interval or did not fall within the range of 0.375 s to 2 s were removed. Since SKNA signals were not rhythmic like ECG signals, the preprocessing of SKNA signals mainly considered the removal of outliers. The interquartile range and an absolute value threshold of 80 μV were used to identify outliers. All values that were not in the range of [Q1-1.5 * IQR, Q3 + 1.5 ^*^ IQR] were removed, where Q1 was the lower quantile and Q3 was the upper quantile. Values outside the absolute value threshold were eliminated. The average SKNA (aSKNA) index was determined by computing the mean of the rectified SKNA signals. ECG and SKNA signals were divided into 5 min and 30 min windows, respectively, with no overlap between windows.

### 2.6. HRV analysis

HRV analysis was performed from three perspectives: the time domain, frequency domain, and nonlinear analysis. The indices of the time domain analysis included the standard deviation of the RR intervals (SDNN), the square root of the mean squared differences of subsequent RR intervals (RMSSD), and the proportion obtained by dividing the number of interval differences of subsequent RR intervals greater than 50 ms by the overall number of the RR intervals (PNN50). The indices of the frequency domain analysis included low frequency (0.04 to 0.15 Hz, LF), high frequency (0.15 to 0.40 Hz, HF), the ratio of low frequency to high frequency (LF/HF), and the ratio of low frequency to the sum of low frequency and high frequency (LF/LF + HF). The nonlinear analysis mainly considered the complexity of RR intervals from the perspective of entropy. The indices of the nonlinear analysis included approximate entropy (ApEn) (Pincus, 1995), sample entropy (Richman and Moorman, 2000; Lake et al., 2002), and fuzzy measure entropy (FuzzyMEn) (Liu et al., 2013). The parameters of ApEn and SampEn were chosen as the dimension *m* = 2 and the tolerance *r* = 0.2. The parameters of FuzzyMEn were chosen as the dimension *m* = 2, the local threshold *r*_l_ = 0.2, the global threshold *r*_g_ = 0.2, the local weight of sequence segments’ similarity *n*_l_ = 3, and the global weight of sequence segments’ similarity *n*_g_ = 2.

### 2.7. Statistical analysis

All data statistics were performed based on SPSS and MATLAB. Shapiro–Wilk test and Kolmogorov–Smirnov test were applied to determine the normality of the data. Continuous data with normal distribution were given as mean ± standard deviation (SD). Otherwise, data were summarized by median (interquartile range [IQR]). Categorical data was given as frequency and percentage. The Levene test was conducted to test the homogeneity of variance. Independent-sample Student’s t-test, Mann–Whitney U test, and Chi-square test were performed to describe the differences between the two subgroups. Student paired t-tests and Wilcoxon signed-rank test were utilized to explore the changes of physiological data in different periods within the group. Pearson correlation coefficient, Spearman correlation coefficient, and least square method were used to analyze the correlation. To investigate the correlation between aSKNA and other indicators, the mean values of segments 1 and 2, 12 and 13, 24 and 25, 35 and 36, 47, and 48 of each index were computed based on the 5 min results, corresponded with the five measurements of SBP and DBP. Two-sided *p* < 0.05 was regarded as significant.

Binary logistic regression was employed for univariate and multivariate analyses to explore the independent risk factors of IDH. In the multivariate analysis, the indicators with *p* < 0.2 in the univariate analysis were included. To more comprehensively explore the underlying association between the indicators of physiological signals and IDH, this study processed the indicators from three dimensions to build multivariate models. Using the 5 min results, we calculated the difference between the last and first segments (DLF), the extreme value of the difference between other segments and the first segments (EOF), and the extreme value of the difference between adjacent segments (EDA). It should be stated that after each calculation of EOF and EDA, the maximum and minimum values were obtained. In univariate analysis, we included the maximum and minimum values of EOF and EDA, respectively, and consider the corresponding extreme values with smaller *p*-values to be included in the multivariate model. To assess the model goodness of fit, the accuracy of the model, the Akaike Information Criterion (AIC), and Omnibus Tests of Model Coefficients were used.

## 3. Results

### 3.1. Participant information

In this study, among the total of 93 patients, 66 subjects had IDH, and 27 subjects did not have that, with an incidence rate of 71.0%. Table 1 displays the baseline data of the total and the two subgroups. The patients in the IDH group were older than those in the nonIDH group (65 [53.3, 69] vs. 54.15 ± 3.1, *p* = 0.039), with higher initial SBP (150.7 ± 2.1 vs. 133.6 ± 2.8, *p* < 0.001). Other characteristics were comparable between the two subgroups, and no significant differences were found. The mean initial SBP was above the 140 mm Hg threshold for hypertension in the IDH group, whereas the mean initial SBP was below this threshold in the nonIDH group.

**Table 1:** Baseline data of the total and the two subgroups.

	Total *N* = 93	IDH *N* = 66	nonIDH *N* = 27	*p* value
Age (year)	62.0[50.0, 69.5]	65.0[52.5, 69.3]	54.2 ± 3.1	**0.039**
Male (n, %)	62 (66.7)	47 (71.2)	15 (55.6)	0.146
Body mass index (kg/m^2^)	22.9 ± 0.4	22.8 ± 0.4	22.1[20.4, 24.4]	0.666
HD duration (year)	3.0[1.4, 6.0]	3.0[1.8, 5.0]	3.0[1.0, 7.0]	0.682
Ultrafiltration (kg)	2.6 ± 0.1	2.5 ± 0.1	2.8 ± 0.2	0.293
Ultrafiltration/Weight (%)	4.1 ± 0.1	3.9 ± 0.2	4.4 ± 0.3	0.155
Pre-HD SBP (mm Hg)	145.7 ± 1.9	150.7 ± 2.1	133.6 ± 2.8	**<0.001**
Pre-HD DBP (mm Hg)	78.3 ± 1.2	79.1 ± 1.4	76.4 ± 2.4	0.331

HD, hemodialysis; IDH, intradialytic hypotension; SBP, systolic blood pressure; DBP, diastolic blood pressure. *p* values for variables with *p*<0.05 are bolded, indicating a significant difference between the two subgroups of indicators.

### 3.2. Changes in BP during HD

**Figure 2:**
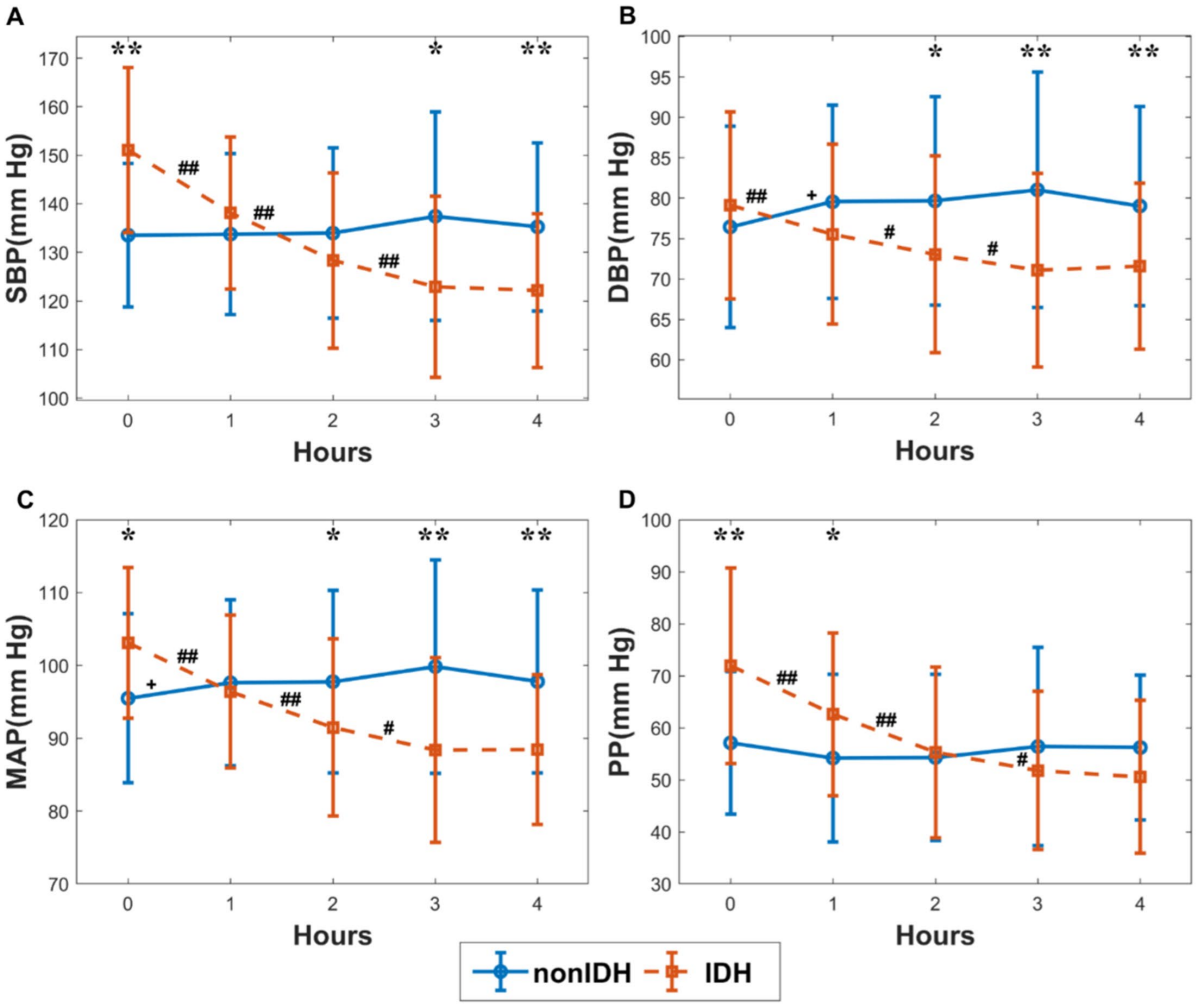
The changes in blood pressure (BP) indicators during hemodialysis (HD) in the subgroups. **(A)** systolic blood pressure (SBP), **(B)** diastolic blood pressure (DBP), **(C)** mean arterial pressure (MAP), **(D)** pulse pressure (PP). The red and blue lines stand for the IDH group and nonIDH group, respectively. ‘#’ and ‘+’ represent statistically significant changes per hour in the IDH group and nonIDH group, respectively. ^##^*p* < 0.001, ^#^*p* < 0.05, ^+^*p* < 0.05. ‘*’ shows statistically significant differences between two subgroups in the same period. ^**^p < 0.001, ^*^p < 0.05. Error bars represent the standard error of the mean (SEM).

The changes in BP indicators during HD in the subgroups are shown in Figure 2. The initial SBP, DBP, MAP, and PP were higher in IDH patients than those in patients without nonIDH, and the differences between the other three indicators were significant except for DBP. On the contrary, the final SBP, DBP, and MAP in IDH patients were lower than those in patients without nonIDH, and the differences in the other three indices were significant except PP. In the IDH group, all four indicators showed a significant decrease during the first 3 hours of HD, but there were no significant changes observed during the last hour, with only slight fluctuations. In the nonIDH group, there was no obvious trend in the changes of BP-related indicators during HD, and the changes were relatively stable, except for the cases of significant rises in DBP and MAP during the first hour. From the perspective of slope changes, the indicators changed most sharply during the first hour, and the degree of change weakened gradually in each subsequent hour.

### 3.3. Changes in heart rate, HRV, and SKNA indices during HD

**Figure 3:**
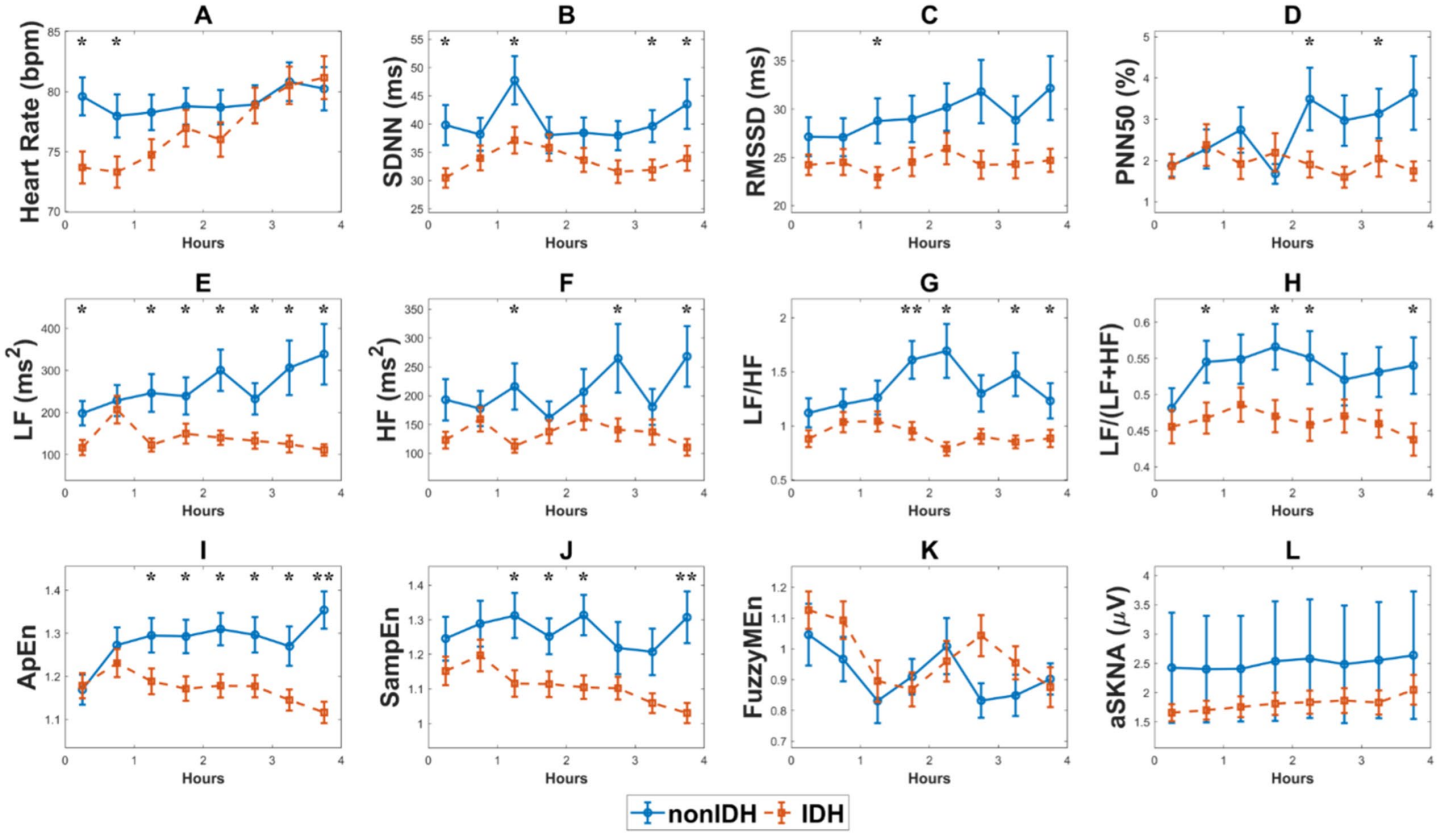
The comparison of heart rate variability (HRV) and skin sympathetic nerve activity (SKNA) indices, as well as heart rate, during hemodialysis (HD) between the two subgroups. **(A)** heart rate, **(B)** the standard deviation of the RR intervals (SDNN), **(C)** the square root of the mean squared differences of subsequent RR intervals (RMSSD), **(D)** the proportion derived by dividing the number of interval differences of subsequent RR intervals greater than 50 ms by the overall number of the RR intervals (PNN50), **(E)** low frequency (LF), **(F)** high frequency (HF), **(G)** LF/HF ratio, **(H)** LF/(LF + HF) ratio, **(I)**. approximate entropy (ApEn), **(J)** sample entropy (SampEn), **(K)** fuzzy measure entropy (FuzzyMEn), **(L)** the average SKNA (aSKNA). The red and blue lines represent the IDH and nonIDH groups, respectively. ‘*’ shows statistically significant differences between two subgroups in the same period. ^**^p < 0.001, ^*^p < 0.05. Error bars represent the standard error of the mean (SEM).

Figure 3 illustrates the comparison of HRV and SKNA indices, as well as heart rate, during HD between the two subgroups. The heart rate in IDH patients was significantly lower than that in patients without nonIDH at the beginning of HD, gradually increased during HD, and was comparable to that of the nonIDH group at the end of HD (Figure 3A). Among the 10 HRV indices in the IDH group, 7 indicators [SDNN, RMSSD, LF, HF, LF/HF, LF/(LF + HF), SampEn] were lower than those of the nonIDH group. At most moments, PNN50 and ApEn were also lower than those of the nonIDH group, while only FuzzyMEn was higher than those of the nonIDH group at most moments. The values of SDNN, RMSSD, and LF/HF increased, while FuzzyMEn decreased in both subgroups. PNN50, LF, HF, LF/(LF + HF), ApEn, and SampEn rose in the nonIDH group and reduced in the IDH group. LF, LF/HF, LF/(LF + HF), ApEn, and SampEn showed good discrimination effects between the two subgroups (Figures 3B-K). For SKNA, aSKNA in the IDH group was lower than that in the nonIDH group, but there was no statistical difference between the two subgroups at each segment. Besides, aSKNA was elevated at end-HD in both groups.

### 3.4. Correlation analysis between aSKNA and other indices

**Figure 4:**
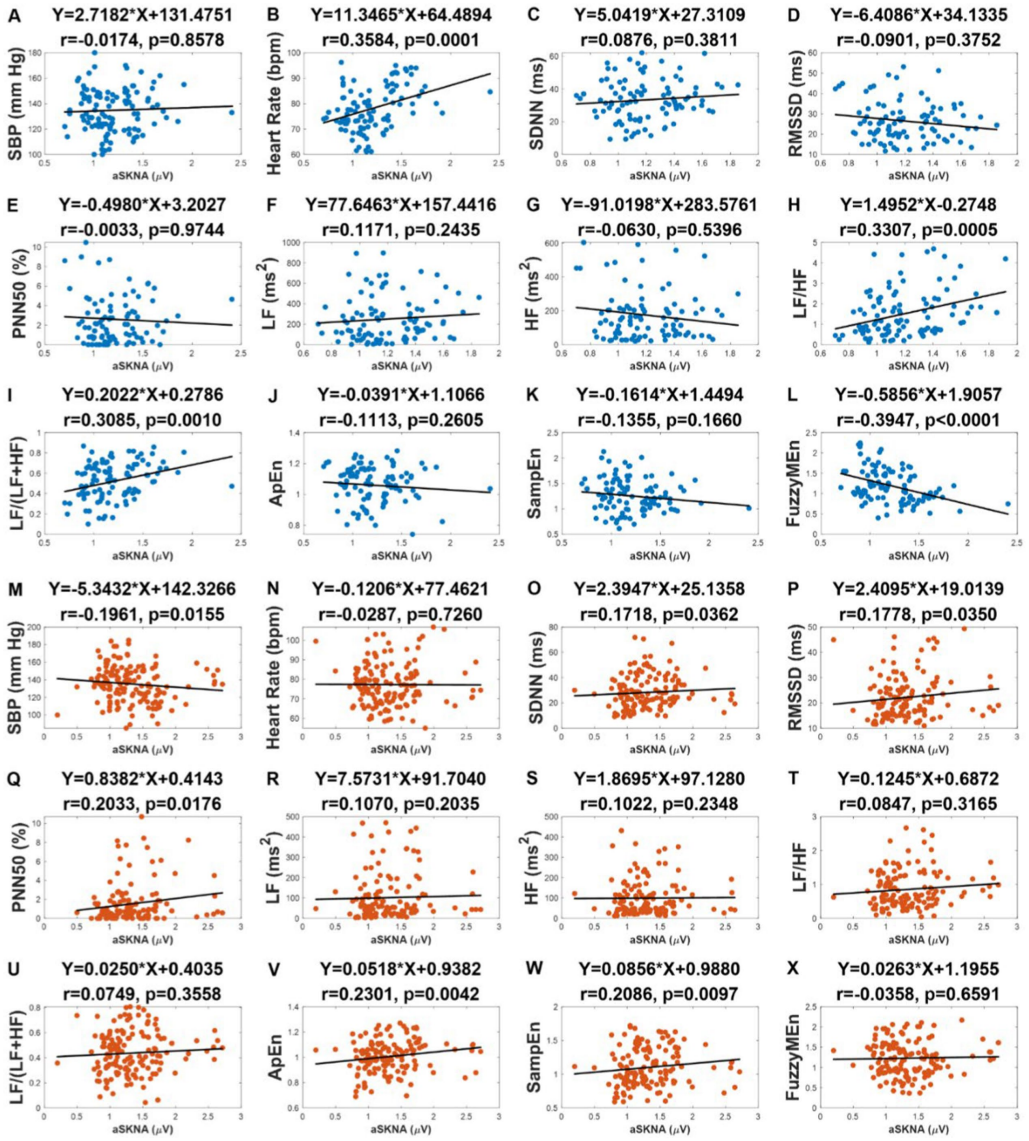
The correlation between the average of skin sympathetic nerve activity (aSKNA) and other physical indicators: systolic blood pressure (SBP), heart rate, heart rate variability (HRV) indices in the intradialytic hypotension (IDH) group and nonIDH group. **(C,O)** the standard deviation of the RR intervals (SDNN); **(D,P)** the square root of the mean squared differences of subsequent RR intervals (RMSSD); **(E,Q)** the proportion derived by dividing the number of interval differences of subsequent RR intervals greater than 50 ms by the overall number of the RR intervals (PNN50); **(F)**, **(R)** low frequency (LF); **(G,S)** high frequency (HF); **(H,T)** LF/HF ratio; **(I,U)** LF/(LF + HF) ratio; **(J,V)** approximate entropy (ApEn); **(K,W)** sample entropy (SampEn); **(L,X)** fuzzy measure entropy (FuzzyMEn). **(A)** to **(L)** represent the results of the nonIDH group, and **(M)** to **(X)** represent the results of the IDH group. The first rows of the figures are titled regression equations obtained by the least squares method, and the second rows are titled correlation coefficients and *p* values.

We examined the correlation between aSKNA and the other physical indicators: SBP, heart rate, and HRV indices in the subgroups, respectively (Figure 4). As it can be seen, there was no correlation between SBP and aSKNA in the nonIDH group, but there was a negative correlation (r = −0.1961, *p* = 0.0155) in the IDH group (Figures 4A,M). A moderate correlation was observed between aSKNA and heart rate in the nonIDH group (*r* = 0.3584, *p* = 0.0001), but no correlation in the IDH group (Figures 4B,N). Interestingly, the HRV indices of the time domain and entropy related to aSKNA in the two subgroups are completely complementary. SDNN (*r* = 0.1718, *p* = 0.0362), RMSSD (*r* = 0.1778, *p* = 0.0350), PNN50 (*r* = 0.2033, *p* = 0.0176), ApEn (*r* = 0.2301, *p* = 0.0042) and SampEn (*r* = 0.2086, *p* = 0.0097) were all positively correlated with aSKNA in the IDH group, while none of these parameters were correlated with aSKNA in the nonIDH group. FuzzyMEn (*r* = −0.3947, *p* < 0.0001) was correlated with aSKNA in the nonIDH group and had no correlation in the IDH group. For parameters in the frequency domain, except LF/HF (*r* = 0.3307, *p* = 0.0005) and LF/(LF + HF) (*r* = 0.3085, *p* = 0.0010) in the nonIDH group, which were positively correlated with aSKNA, there was no correlation in other cases.

### 3.5. Establishment of IDH risk factor model

Binary logistic regression was utilized to establish models. Firstly, univariate analysis was used to analyze the influence of baseline data on IDH. The results of the univariate analysis using the baseline data are reported in Table 2. Among these characteristics, variables with *p* < 0.2 in the univariate analysis, including age, sex, ultrafiltration/weight, and SBP before HD, were integrated into the multivariate analysis.

**Table 2:** The results of the univariate analysis using the baseline data for evaluating intradialytic hypotension (IDH).

Variable	OR(95% CI)	*p* value
Age	1.042 (1.007, 1.078)	**0.018**
Sex	0.543 (0.216, 1.368)	**0.195**
Body mass index	0.994 (0.882, 1.121)	0.922
HD duration	0.974 (0.876, 1.082)	0.619
Ultrafiltration	0.772 (0.477, 1.248)	0.291
Ultrafiltration/Weight	0.000 (0.000, 13232.122)	**0.155**
Pre-HD SBP	1.071 (1.033, 1.110)	**<0.001**
Pre-HD DBP	1.019 (0.981, 1.060)	0.328

Pre-HD, before hemodialysis; SBP, systolic blood pressure; DBP, diastolic blood pressure; OR, odds ratio; CI, confidence interval. *p* values were obtained from logistic regression, and *p* values for variables with *p*<0.2 are bolded.

Then, to better evaluate the impact of HRV and SKNA indicators on IDH, we processed the indicators in three different ways and obtained the results of DLF, EOF, and EDA. The results of the univariate models and multivariate models using these calculated variables are listed in Table 3. For HRV indices, DLF showed that most HRV indicators were integrated into the multivariate analysis, and the multivariate model performed best, with the highest accuracy (77.4) and lowest AIC (274.268). By contrast, the minimal values of EOF and EDA for aSKNA were statistically significant in univariate analysis and were included in subsequent multivariate analysis.

**Table 3:** The results of univariate analysis and multivariate analysis of heart rate variability (HRV) and skin sympathetic nerve activity (SKNA) indices for evaluating intradialytic hypotension (IDH).

			Univariate analysis			Multivariate analysis		
			DLF	EOF	EDA	Model 1	Model 2	Model 3
# *p* values	HRV	Heart Rate	**0.009**	**0.015**^−^	0.662^+^	**0.016**	**0.049**^−^	–
		SDNN	0.373	0.315^+^	0.558^+^	–	–	–
		RMSSD	**0.141**	0.204^+^	0.560^−^	0.236	–	–
		PNN50	**0.141**	**0.132**^**+**^	0.321^+^	0.215	**0.147**^**+**^	–
		LF	0.333	0.795^+^	0.861^+^	–	–	–
		HF	**0.151**	0.329^+^	0.384^+^	**0.060**	–	–
		LF/HF	**0.011**	**0.085**^**+**^	**0.120**^−^	**0.016**	0.386^**+**^	**0.135**^−^
		LF/(LF + HF)	**0.109**	0.208^−^	0.365^+^	**0.113**	–	–
		ApEn	0.460	0.297^−^	0.303^+^	–	–	–
		SampEn	**0.186**	0.775^+^	0.578^−^	0.480	–	–
		FuzzyMEn	0.505	**0.165**^−^	**0.035**^**+**^	–	0.694^−^	**0.038**^**+**^
	SKNA	aSKNA	0.768	**0.009**^−^	**0.039**^−^	–	–	–
Constant			–	–	–	**0.021**	**0.044**	0.968
Accuracy			–	–	–	77.4	73.1	69.9
AIC			–	–	–	274.268	286.839	290.921
*p value			–	–	–	0.010	0.024	0.028

DLF, the difference between the last and first segments; EOF, the extreme value of the difference between other segments and the first segment; EDA, the extreme value of the difference between adjacent segments; SDNN, the standard deviation of the RR intervals; RMSSD, the square root of the mean squared differences of subsequent RR intervals; PNN50, the proportion derived by dividing the number of interval differences of subsequent RR intervals greater than 50 ms by the overall number of the RR intervals; LF, low frequency; HF, high frequency; LF/HF, LF/HF ratio; LF/(LF + HF), LF/(LF + HF) ratio; ApEn, approximate entropy; SampEn, sample entropy; FuzzyMEn, fuzzy measure entropy; aSKNA, the average SKNA; AIC, Akaike Information Criterion. In extreme value calculation, the maximum value and the minimum value are included, and ‘^+^’ and ‘^−^’ represent the maximum value and the minimum value, respectively. #*p* values were obtained from logistic regression, and *p* values for variables with *p* < 0.2 are bolded. ^*^p values in the last row were obtained from Omnibus Tests of Model Coefficients. ^*^p < 0.05 indicates that the model is overall significant.

Finally, the baseline data and SKNA indices with p < 0.2 in the univariate analysis and HRV indices from the HRV multivariate Model 1 were integrated into the multivariate model for comprehensive analysis. Five models with different combinations of variables are reported in Table 4. Compared with model 1, models 2, 3, and 4 were optimized by adding HRV and SKNA parameters. Model 3 had the highest accuracy (84.9) and lowest AIC (250.356), which was also superior to the HRV multivariate Model 1 (Table 3). We found that higher SBP before HD [odds ratio (OR) 1.076; 95% confidence interval (CI) 1.031–1.124, *p* = 0.001], heart rate-DLF [OR 1.101; 95% CI 1.022–1.187, *p* = 0.012], and aSKNA-EOF [OR 2.908; 95% CI 1.210–6.989, *p* = 0.017], and lower LF/HF-DLF [OR 0.209; 95% CI 0.049–0.885, *p* = 0.034] were four independent indicators for IDH (Table 5).

**Table 4:** The results of the different multivariate models for predicting intradialytic hypotension (IDH).

		Model 1	Model 2	Model 3	Model 4	Model 5
#*p* value	Pre-HD SBP	**< 0.001**	**0.001**	**0.001**	**0.001**	–
	Age	0.085	0.577	0.874	0.706	–
	Ultrafiltration/Weight	0.297	0.133	0.087	0.110	–
	Sex	0.265	0.551	0.806	0.584	–
	Heart Rate-DLF	–	**0.021**	**0.012**	**0.024**	**0.015**
	LF/HF-DLF	–	**0.039**	**0.034**	**0.037**	**0.024**
	HF-DLF	–	0.147	0.169	0.158	**0.038**
	LF/(LF + HF)-DLF	–	0.214	0.186	0.210	0.131
	RMSSD-DLF	–	–	–	–	0.130
	PNN50-DLF	–	–	–	–	0.184
	SampEn-DLF	–	–	–	–	0.569
	aSKNA-EOF^**−**^	–	–	**0.017**	–	**0.010**
	aSKNA-EDA^**−**^	–	–	–	0.132	–
Constant		**0.001**	**0.013**	**0.030**	**0.019**	**0.002**
Accuracy		77.4	81.7	84.9	82.8	78.5
AIC		271.835	255.684	250.356	253.349	265.998
*p value		<0.001	<0.001	<0.001	<0.001	<0.001

Pre-HD, before hemodialysis; SBP, systolic blood pressure; LF, low frequency; HF, high frequency; LF/HF, LF/HF ratio; LF/(LF+HF), LF/(LF+HF) ratio; RMSSD, the square root of the mean squared differences of subsequent RR intervals; PNN50, the proportion derived by dividing the number of interval differences of subsequent RR intervals greater than 50 ms by the overall number of the RR intervals; SampEn, sample entropy; aSKNA, the average of skin sympathetic nerve activity; AIC, Akaike Information Criterion. ‘-DLF’ denotes the difference between the last and first segments of the calculated variable. ‘-EOF^**−**^’ denotes the minimum value of the difference between other segments and the first segment of the calculated variable. ‘-EDA^**−**^’ denotes the minimum value of the difference between adjacent segments of the calculated variable. #p values were obtained from logistic regression, and p values for variables with *p* < 0.05 are bolded. *p values in the last row were obtained from Omnibus Tests of Model Coefficients. *p < 0.05 indicates that the model is overall significant.

**Table 5:** The optimal model for predicting intradialytic hypotension (IDH) based on logistic regression.

	OR(95% CI)	*p* value
Pre-HD SBP	1.076 (1.031, 1.124)	**0.001**
Age (per 1 year)	1.004 (0.956, 1.054)	0.874
Ultrafiltration/Weight	0.000 (0.000, 1606.431)	0.087
*Sex*		
Male	1.000	–
Female	0.844 (0.218, 3.271)	0.806
Heart Rate-DLF	1.101 (1.022, 1.187)	**0.012**
LF/HF-DLF	0.209 (0.049, 0.885)	**0.034**
HF-DLF	1.001 (1.000, 1.002)	0.169
LF/(LF + HF) -DLF	247.191 (0.070, 869089.763)	0.186
SKNA-EOF-	2.908 (1.210, 6.989)	**0.017**
Constant	0.001	**0.030**

Pre-HD, before hemodialysis; SBP, systolic blood pressure; LF, low frequency; HF, high frequency; LF/HF, LF/HF ratio; LF/(LF+HF), LF/(LF+HF) ratio; aSKNA, average of skin sympathetic nerve activity; OR, odds ratio; CI, confidence interval. ‘-DLF’ denotes the difference between the last and first segments of the calculated variable. ‘EOF-’ denotes the minimum value of the difference between other segments and the first segment of the calculated variable. *p* values for variables with *p* < 0.05 in the logistic regression are bolded.

## 4. Discussion

Time and frequency domain analysis of HRV was widely utilized in previous IDH-related studies. However, this traditional method ignores the nonlinear dynamical information on heart rate (Denton et al., 1990) and lacks a more intuitive description of SNA (Kusayama et al., 2020b). We implemented the methods for the entropy of HRV and SKNA to explore the physiological mechanism of IDH, which described the nonlinear and dynamic changes of the ANS in IDH. Two distinct response patterns of the ANS during HD were observed in the two subgroups, and the IDH group showed worse ANS activity and ability to cope with the stimulation. Higher initial SBP, the DLF of heart rate, and the EOF of aSKNA, as well as the lower DLF of LF/HF were found to be independent indicators for IDH.

### 4.1. ANS patterns revealed by HRV indices

The overall level of HRV indices, excluding FuzzyMEn, was lower in the IDH group than that in the nonIDH group. Reduced HRV is a remarkable predictor of symptoms and death of a wide broad spectrum of diseases, especially cardiovascular diseases (Sessa et al., 2018; Fang et al., 2020). LF/HF, which describes the balance of SNA and parasympathetic nervous activity (PNA), increased in both subgroups, consistent with the previous study (Chang et al., 2016; Park et al., 2019). However, the same outcomes indicate different ANS patterns, mainly due to increased LF in nonIDH patients and decreased HF in IDH patients. Lower HF and reduced HF show suppressed PNA in the IDH group, which also points to a poor cardiac prognosis (Algra et al., 1993).

Moreover, inconsistent interpretations of indices in previous studies affected the credibility of HRV. LF was initially interpreted to characterize SNA. Nonetheless, accumulating evidence demonstrates that LF represents a nonlinear interaction between SNA and PNA (Billman, 2013; Chang et al., 2016). Although LF/HF indirectly represents the intensity of SNA through the balance of ANS, when the ANS function is weakened to a certain extent, LF/HF will lose its significance (Billman, 2013). This hypothesis is confirmed by the evidence that LF/HF and aSKNA were positively correlated in the nonIDH group, but this correlation disappeared in the IDH group with weaker ANS function. Consequently, in the assessment of patients with impaired ANS function, such as those with IDH, HRV may not provide an accurate evaluation of SNA, and the obtained results should be interpreted with caution.

### 4.2. Evaluation of entropy and SKNA during HD

ApEn and SampEn and FuzzyMEn describe the regularity of RR intervals. ApEn is used to address short-time noisy signals, with strong robustness (Pincus, 1991). SampEn solves the self-matching of the template in ApEn and is less dependent on data length (Richman and Moorman, 2000). FuzzyMEn introduces the fuzzy measure of variable similarity and considers both local and global similarity comprehensively to have better performance in short-time series processing (Liu et al., 2013). The lower values of ApEn and SampEn in the IDH group exhibit that patients who had higher regularity of heart rate may be prone to a higher risk of cardiovascular diseases (Fleisher et al., 1993; Mäkikallio et al., 1998; Rajendra Acharya et al., 2006; Shin et al., 2006). Besides, it was found that ApEn and SampEn showed better discrimination ability for IDH in comparing the differences in index values between the two subgroups (Figures 3I-K), indicating that ApEn and SampEn can better characterize the difference in ANS function. But only FuzzyMEn was included in model 3 of HRV when determining the risk factors (*p* < 0.05, Table 3). This illustrates that introducing a global perspective helps predict the tendency of IDH over time. FuzzyMEn showed better performance on the 5-min scale compared to the 30-min scale, confirming the superiority of FuzzyMEn in the processing of short-time sequences.

As an emerging tool for assessing SNA, SKNA overcomes the limitation that HRV needs to be based on sinus rhythm and elevates time resolution to the level of seconds. During HD, aSKNA increased in both subgroups, similar to LF/HF, suggesting sympathetic activation, consistent with the expected feedback of compensatory mechanism triggered by increased ultrafiltration, which was in line with previous studies (Park et al., 2019; Zhang et al., 2022). Lower aSKNA in the IDH group indicated that patients had insufficient sympathetic activation (Converse et al., 1992). Moreover, aSKNA was positively correlated with heart rate in the nonIDH group, but this relationship disappeared in the IDH group. The loss might imply worse neurologic recovery, which was also observed in individuals receiving targeted temperature control (Kutkut et al., 2021). aSKNA was negatively correlated with SBP in the IDH group, but this relationship was lost in the IDH group, which revealed a worse systemic ability to resist the loss of volume in IDH patients. In the IDH group, not only the activation of SNA was insufficient, but also other physiological mechanisms failed to resist the reduction of BP.

### 4.3. Risk factors for IDH

DLF, EOF, and EDA methods were used to process HRV and SKNA indices in the establishment of the IDH multivariate model. DLF model of HRV performed best, indicating that the difference in HRV values before and after dialysis better described the occurrence of IDH. In the final multivariate model comparison, the two iterations from model 1 to model 3 indicated that adding HRV indices and SKNA index based on clinical baseline information helped assess IDH, respectively. The comparison of models 3 and 4 showed that the maximum change in SKNA from the initial value could better distinguish IDH. That is, the changes in SNA during HD affect the development of IDH relative to the baseline. Model 5 was introduced to illustrate the importance of baseline data in the model.

In the final assessment model of IDH, higher initial SBP, the DLF of heart rate, and the EOF of aSKNA, as well as the lower DLF of LF/HF became the risk factors for IDH. Higher initial BP promotes IDH which is consistent with previous study (Chang et al., 2016). The mean initial SBP in IDH patients was above the diagnostic criteria of hypertension, which is 140 mmHg, whereas that in nonIDH patients were normal. On one hand, we speculate that hypertension may be related to greater weight gain when not receiving HD but with inadequate dialysis because of an unexpected weight gain. On the other hand, we think that IDH patients initially may have over-activated sympathetic nerves, which are more susceptible to hypertension (Masuo et al., 2010), resulting in vascular overload and pathological changes (Cachofeiro et al., 2009), but abnormal SNA gradually decreases with the increase of the HD duration (Masuo et al., 2010), even lower than other HD patients. Therefore, patients are unable to further raise peripheral vascular resistance in response to the increase in SNA, resulting in IDH (Dubin et al., 2011). In addition, LF is nonlinearly regulated by the SNA and PNA, as well as other factors, with the effect of PNA being approximately twice as strong as that of SNA (Randall et al., 1991). That is, LF/HF is more sensitive to PNA at lower SNA. Thus, we hypothesized that the lower DLF of LF/HF directed to the depressed PNA or other factors in IDH patients, which are different from the depressed SNA generally believed.

### 4.4. Limitation

It should be noted that this study still has several limitations. First of all, this research was conducted in a single center, and the sample size of patients was small, so the data may be biased. Secondly, the lack of clinical symptoms and interventions reduced the physiological differences between IDH and nonIDH patients, making it more difficult to identify IDH. What is more, physical data during HD were collected only once for each patient, and no long-term follow-up was formed, so there may be some contingency in the results. Finally, although the SKNA assessment achieved satisfactory results in this study, the specific physiological mechanism of SKNA is still unclear, and the underlying relationship between SKNA and HRV indicators needs to be further studied.

## 5. Conclusion

In this study, a portable, noninvasive, and high-frequency electrophysiological acquisition device was used to collect ECG and SKNA signals of patients during HD, which were combined with baseline data to evaluate ANS function during HD. Compared with previous studies, this study introduced the entropy of HRV and SKNA methods to provide a nonlinear and dynamic perspective of ANS function assessment and investigated the underlying physiological mechanism of IDH. We found different patterns in response to plasma loss between IDH patients and nonIDH patients, and the IDH group exhibited worse ANS function. In addition, we found that higher initial SBP, the DLF of heart rate and the EOF of aSKNA, and the lower DLF for LF/HF were independent factors of IDH. The SKNA showed good performance in both group comparison and model evaluation in this study. Although the entropy of HRV was not integrated into the final multivariate model, the nonlinear information it provided deserved further exploration.

## Data availability statement

The original contributions presented in the study are included in the article/supplementary materials, further inquiries can be directed to the corresponding authors.

## Ethics statement

The studies involving human participants were reviewed and approved by the Ethics Committee of the First Affiliated Hospital of Nanjing Medical University. The patients/participants provided their written informed consent to participate in this study.

## Author contributions

JiayL, YX, CC, and CL contributed to the conception and design of the study. JiayL and JianL processed the signals, performed the statistical analysis, and wrote the manuscript. YX provided the devices and processed the signals. YZ, CC, and JW collected the data and organized the database. JianL and CL contributed to the guidance of the whole study. All authors contributed to the article and approved the submitted version.

## Funding

This work was supported by the National Natural Science Foundation of China (62171123, 62071241, 62201144, and 62211530112), and the Natural Science Foundation of Jiangsu Province of China (BK20192004).

## Conflict of interest

The authors declare that the research was conducted in the absence of any commercial or financial relationships that could be construed as a potential conflict of interest.

## Publisher’s note

All claims expressed in this article are solely those of the authors and do not necessarily represent those of their affiliated organizations, or those of the publisher, the editors and the reviewers. Any product that may be evaluated in this article, or claim that may be made by its manufacturer, is not guaranteed or endorsed by the publisher.
